# High-Resolution Low-Sidelobe Waveform Design Based on HFPFM Coding Model for SAR

**DOI:** 10.3390/s25237383

**Published:** 2025-12-04

**Authors:** Yu Gao, Guodong Jin, Xifeng Zhang, Daiyin Zhu

**Affiliations:** 1 Key Laboratory of Radar Imaging and Microwave Photonics, Ministry of Education, Nanjing University of Aeronautics and Astronautics, Nanjing 211116, China; gao-yu@nuaa.edu.cn (Y.G.); zhangxifeng@nuaa.edu.cn (X.Z.);; 2 Shenzhen Research Institute, Nanjing University of Aeronautics and Astronautics, Shenzhen 518057, China

**Keywords:** low-sidelobe, high-resolution, SAR imaging, HFPFM coding model, gradient descent, waveform optimization design

## Abstract

Radar waveform design is an important approach to radar system performance enhancement. For a long time, synthetic aperture radar (SAR) systems have utilized linear frequency modulation (LFM) waveforms as transmitted signals and have relied on window functions to suppress sidelobes. However, this approach significantly degrades system signal-to-noise ratio (SNR) and resolution. Nonlinear frequency modulation (NLFM) waveforms can suppress sidelobes without SNR loss and have been widely applied in the SAR field in recent years. Nonetheless, they still cannot completely avoid resolution loss. To address this, this article, based on an advanced High-Freedom Parameterized Frequency Modulation (HFPFM) coding model, constructs a waveform sidelobe optimization model constrained by mainlobe widening and solves it using a gradient descent method. Through detailed experiments, we found that the optimized waveform, compared to the LFM waveform, can reduce sidelobes by more than 9 dB without widening the mainlobe, thereby simultaneously avoiding the resolution and SNR losses caused by window function weighting. In addition, this optimization method can efficiently and rapidly optimize all parameters simultaneously using only matrix multiplication and fast Fourier transform (FFT)/inverse fast Fourier transform (IFFT). The SAR point target imaging simulation results verify that the optimized waveform can clearly image weak targets near strong targets, which proves the effectiveness of the proposed method.

## 1. Introduction

SAR is an airborne imaging sensor used for terrestrial mapping and is regarded as one of the most important tools in remote sensing. SAR transmits signals with a large time-bandwidth product, and high range resolution is obtained after pulse compression. Due to the distributed target characteristics of SAR, the interaction between strong and weak image targets is significant (especially in urban areas). Therefore, sidelobe suppression is a primary research focus of SAR systems.

High sidelobes adversely affect radar target detection in two primary ways: first, weak targets may be obscured by sidelobes generated by strong targets, leading to missed detections; second, the diffusion of target energy within adjacent range resolution cells may result in the appearance of false targets. In SAR systems, LFM waveforms are the most commonly used transmitted signals. However, their Fourier transform yields a sinc function with a peak sidelobe ratio (PSLR) of −13 dB. The PSLR in SAR systems must be less than this value to prevent weak targets from being drowned out by adjacent strong targets. For SAR systems, an acceptable PSLR should be below −20 dB [1]. Therefore, the design of low sidelobe waveforms holds significant importance for SAR signal processing.

The primary existing methods for reducing sidelobes include three approaches: optimizing mismatch filters [2], designing transmitted waveforms [3], and jointly designing transmitted waveforms and mismatch filters [4]. The purpose of a matched filter is to maximize the SNR, but it may generate significant sidelobes. These sidelobes can be reduced using a mismatched filter. The first method, which is the design of mismatched filters, primarily involves the *L_p_*-norm Lagrange multiplier method for minimizing sidelobe energy [2], weighted window functions [5], adaptive pulse compression [6], modified spatially variant apodization (SVA) algorithms [7], and sidelobe suppression formulas based on second-order cone programming (SOCP) [8]. Mismatch filters are simple to implement and require no modifications to the transmitted waveforms, but they result in some processing gain loss [9]. The design of mismatched filters aims to reduce the output signal’s PSLR by optimizing the filter parameters. The basic principle is to sacrifice a certain degree of the matched filter’s output SNR in exchange for a reduction in sidelobe levels [10], which greatly increases the power budget of spaceborne SAR and decreases the efficiency of radar signal processing. Therefore, improving the processing efficiency of radar systems is an urgent issue that needs to be addressed. Consequently, the design of the transmitted waveforms has been popular.

The second method, which is the transmitted waveform design, is a key approach to reducing PSLR. By carefully designing the parameters and structure of the transmitted waveform, radar echoes can exhibit low sidelobe characteristics. There are two primary types of low-sidelobe transmitted waveforms: those with low global sidelobes and those with low local sidelobes. Global low-sidelobe transmitted waveforms exhibit reduced sidelobe levels across the entire signal range, whereas local low-sidelobe waveforms achieve low sidelobes within specific regions of interest (ROI) while potentially accepting higher sidelobe levels in other areas. The primary methods for designing transmitted waveforms with low global sidelobes include the cyclic algorithm new (CAN) [11], directly minimizing the integrated sidelobe level (ISL) of the autocorrelation [12], and the heuristic method based on coordinate descent (CD) [13], etc. [3], which applies the alternating direction method of multipliers (ADMM) to synthesize the probing sequences. Regarding the design of waveforms for local low-sidelobes, refs. [14,15,16,17] focus on unimodular waveforms design that optimize the ambiguity function (AF) within ROI, i.e., methods based on local AF optimization. References [11,18] present approaches based on local autocorrelation optimization. Low sidelobes at specific locations do not guarantee lower PSLR for the waveform. Waveforms with low PSLR hold greater practical significance, as lower PSLR typically results in reduced false alarm probability [19]. The transmitted waveform design offers high flexibility by suppressing sidelobes at the source.

Regarding the joint design of transmitted waveforms and mismatch filters, which is the third method, ref. [4] developed an efficient algorithm based on Dinkelbach’s algorithm and majorization minimization (MM) method to minimize the sidelobes. Reference [20] proposes an optimization method that maximizes the signal-to-clutter-plus-interference ratio. Reference [21] presents a method that minimizes the peak sidelobe level (PSL) of the range sidelobes after mismatched filtering with a constraint of SNR loss with respect to that after matched filtering. Reference [9] introduces a gradient descent-based joint design method for simultaneous optimization waveforms and their filters, aiming to minimize PSL. There are also some other optimization algorithms, such as [22,23]. Joint design of the transmitted waveform and mismatch filter enables the globally optimal reduction in PSLR, but will lose SNR. This article proposes optimizing the waveform from the perspective of transmitted waveform design under the matched filtering framework, which will not lose SNR.

Unlike the linear time–frequency relationship of LFM, the time–frequency relationship of NLFM is approximately ‘S’-shaped. Compared to LFM waveforms, NLFM waveforms construct power spectral density (PSD) by altering the time–frequency relationship of the signal, allowing for a reduction in PSLR without sacrificing the SNR. To the best of our knowledge, most methods for generating NLFM waveforms are based on the principle of stationary phase (POSP) [24,25,26], which links the PSD to the chirp rate at each frequency, thus shaping a spectrum that meets the requirements. Due to the Fourier transform relationship between PSD and the autocorrelation function, obtaining waveforms with a high-quality spectrum yields an ideal autocorrelation function. Unfortunately, the aforementioned methods for generating NLFM waveforms are based on the POSP use window functions for spectral shaping, which can lead to mainlobe broadening and affect the resolution of SAR systems. Therefore, designing high-resolution low-sidelobe waveforms is important for enhancing the performance of SAR systems. This article proposes a waveform optimization method that generates lower sidelobes and a narrower mainlobe without sacrificing the SNR.

Phase-coded waveforms have the advantage of a parameterized coding structure, but their instantaneous phase is variable, resulting in high sidelobes and making them unsuitable for high-power systems. Frequency-modulated signals are suitable for high-power transmitters, whereas other types of waveforms may cause significant distortion. The polyphase-coded frequency modulation (PCFM) waveform exhibits a continuous phase function; however, their spectral range is controlled by frequency template errors (FTE), which greatly increase the complexity of the design. NLFM waveforms can precisely define spectral ranges by setting the range of instantaneous frequency, but they lack a parameterized coding structure, limiting their long-term application. To that end, the recently proposed HFPFM coding model in [27] combines the advantages of these waveforms: it features a parameterized coding structure, a continuous phase function, and a precise control over the spectral range.

This article optimizes the HFPFM-coded waveform using gradient descent methods, which constructs a waveform sidelobe optimization model constrained by mainlobe widening and solves it using a gradient descent method. Through detailed experiments, we found that the optimized waveform, compared to LFM, can reduce PSLR by more than 9 dB without widening the mainlobe; compared to NLFM waveforms with the same PSLR, it has a smaller mainlobe width, resulting in an equivalent improvement of nearly 15% in resolution without increasing the PSLR value. Therefore, the optimization method we propose can achieve a waveform with a smaller mainlobe and a lower PSLR, resulting in a high-resolution low-sidelobe waveform. Compared with NLFM waveforms, the waveform we designed has the characteristic of parametric coding, which is more conducive to the optimized design of the waveform. It is precisely because the HFPFM coding model we use has a parametric encoding ability that we are able to construct an analytical signal model, solve it using the gradient descent method, and obtain better results than the NLFM waveform [28], which lacks the parametric encoding capability. In Section 3.2.2, we present the results of a comparison between the algorithm proposed in this article and the algorithm in [28]. The results show that when using the LFM waveform as the initial waveform, the method proposed in this article can reduce the PSLR to −22.9607 dB, while the method proposed in [28] can only reduce the PSLR to −18.51 dB. When using the NLFM waveform as the initial waveform, to achieve the same PSLR, the mainlobe width of the method proposed in this article is smaller than that of the method in [28], effectively improving the resolution by nearly 4%. The gradient descent method can update all parameters simultaneously and can be effectively implemented using only matrix multiplication and FFT/IFFT. In addition, we applied this method to generate a waveform with extremely low sidelobes (−82.8 dB). The SAR point target imaging simulation results verify that the optimized waveform can clearly image weak targets near strong targets, which proves the effectiveness of the proposed method.

Reference [27] only proposed the HFPFM encoding model and does not involve algorithms for waveform optimization. The advantage of this article compared to reference [27] is that it constructs a sidelobe optimization model with a mainlobe width constraint and uses a gradient descent algorithm for optimization. References [29,30] also use the gradient descent algorithm to optimize the waveform. The main difference between this article and Refs. [29,30] is the parameterized model. References [29,30] both use the parameterization of PCFM. In contrast, this article uses the HFPFM coding model for signal parameterization. In addition, compared with the gradient descent methods in Refs. [29,30], this article uses a penalty function to constrain the spectral range, thereby precisely controlling the range of the spectrum. This can reduce out-of-band energy and decrease the loss of transmission power, effectively enhancing the transmission power.

Compared with CAN, ADMM, and CD, the novelty of this method lies in the fact that the designed waveform has a continuous phase and strict spectral range constraints. CAN, ADMM, and CD are phase-coded constant modulus sequence design methods, which can produce phase jumps of arbitrary shapes, whereas the method proposed in this article belongs to the frequency-modulated waveform design approach, producing waveforms with a continuous phase.

Compared with the design method of joint waveforms and mismatched filters, this method can reduce sidelobes without sacrificing SNR. A matched filter maximizes the SNR of the output signal, while a mismatched filter reduces sidelobes at the expense of the signal’s SNR. The design method of joint waveforms and mismatched filters essentially still incurs SNR loss. SAR is a long-range, high-resolution radar system, especially spaceborne SAR, where energy in space is very limited. If the SNR is lost, it will affect image sensitivity. Therefore, we aim to study methods of reducing waveform sidelobes within the framework of matched filters. The advantage of this method over the design method of joint waveforms and mismatched filters is that it can reduce sidelobes without sacrificing SNR. In addition, all derivations in this method are based on a mathematical analytical model and do not impose restrictions on parameters such as waveform duration or pulse width; hence, there are no constraints for the establishment of improvements.

The rest of this article is organized as follows: Section 2 presents the optimization model based on HFPFM coding; Section 3 describes the implementation of the optimization model; Section 4 provides the results of point target imaging simulations; and Section 5 concludes this article.

## 2. Optimization Model Based on HFPFM Coding Model

### 2.1. HFPFM Waveform Implementation

**Figure 1:**
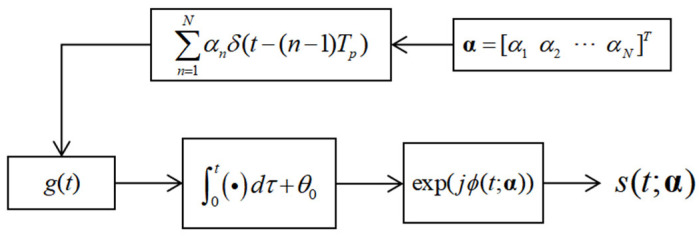
Brief description of the HFPFM waveform formation process.

In brief, the HFPFM waveform implementation takes a vector of N parameters $\alpha ={\left[\alpha_{1}\;\alpha_{2}\;\cdots \;\alpha_{N}\right]}^{T}$, where ${\left(•\right)}^{T}$ is the vector transpose operation, and uses them to weight a train of N impulses separated in time by $T_{P}$. The maximum value of $\alpha_{n}$ (*n* = 1, 2,…, N) is B/2, for B is the bandwidth of an HFPFM-coded waveform and the minimum value of $\alpha_{n}$ (*n* = 1, 2,…, N) is −B/2. This weighted impulse train is convolved with g(t), which is the frequency shaping filter and has a time content of $\left[0,T_{p}\right]$. It is then integrated to form a continuous phase function of time: ϕ(t;α). Subsequently, this phase function is exponentialized to produce a complex baseband waveform with a pulse width of $NT_{p}$. This process is illustrated in Figure 1.

The process in Figure 1 can be succinctly written as

(1) $$\begin{array}{c} s\left(t;\alpha \right)=\exp\left\{j\left(\int_{0}^{t}g\left(\tau \right)*\left[\sum_{n=1}^{N}\alpha_{n}\delta \left(t-\left(n-1\right)T_{p}\right)\right]d\tau \right)\right\}\\ \;\;\;\;=\exp\left(j\phi \left(t;\alpha \right)\right) \end{array}$$

where ∗ represents a convolution operation. The phase components in (1) can be equivalently expressed as

(2) $$\phi (t;\alpha )=\sum_{n=1}^{N}\alpha_{n}b_{n}\left(t\right)$$

where the $b_{n}\left(t\right)$ can be expressed as

(3) $$b_{n}\left(t\right)=\int_{0}^{t}g\left(\tau -\left(n-1\right)T_{p}\right)d\tau$$

It is an integral of the shaping filter and the delay-shifted by an integer multiple of $T_{p}$. Here is an example. For the HFPFM waveform, g(t) is a rectangular shaping filter, and its amplitude is $1/T_{p}$. The *n*th basis function is the ramp function of the delay-shifted:

(4) $$b_{n}\left(t\right)=\left\{\begin{array}{ll} 0, & 0\leq t\leq \left(n-1\right)T_{p}\\ \left(t-\left(n-1\right)T_{p}\right)/T_{p}, & \left(n-1\right)T_{p}\leq t\leq nT_{p}\\ 1, & nT_{p}\leq t\leq NT_{p} \end{array}\right.$$

Therefore, the HFPFM waveform’s phase consists of a linear combination of these N continuous basis functions, each of which is weighted by the corresponding HFPFM parameter $\alpha_{n}$.

A discretized HFPFM-coded waveform can be evaluated by a single matrix/vector multiplication. Consequently, a discrete HFPFM-coded waveform of length $M\left(\geq N\right)$ can be represented as

(5) $$s=\exp\left(jB\alpha \right)$$

where B is an M×N matrix consisting of a sampled version of N basic functions of the length M.

In order to facilitate subsequent optimizations, we set α=x•B, where B is the bandwidth of the signal. Then, we rewrite Formula (5) in the following form:

(6) $$s=\exp\left(jBxB\right)$$

Thus, the maximum value of x is 0.5 and the minimum value is −0.5.

### 2.2. Optimization Model

As we all know, for a signal $s\left(t\right)$, its continuous autocorrelation can be written as

(7) $$r\left(\tau \right)=\int_{-\infty }^{\infty }s\left(t\right)s^{*}\left(t+\tau \right)dt$$

where ${\left(•\right)}^{*}$ denotes complex conjugation. The two most important metrics are the integrated sidelobe level ratio (ISLR), which compares the total energy of the autocorrelation sidelobe regions with the total energy in the mainlobe region, and the PSLR, which compares the peak level of the maximum value of the autocorrelation sidelobes with the value at the peak of the mainlobe. The PSLR and ISLR can be summarized as [29]

(8) $$G={\left(\frac{\int_{\Delta t}^{T}{\left|r\left(\tau \right)\right|}^{p}d\tau }{\int_{0}^{\Delta t}{\left|r\left(\tau \right)\right|}^{p}d\tau }\right)}^{2/p}$$

The value of p can be 2≤p<∞. The p value between 2 and ∞ provides a measure between ISLR and PSLR, and large and limited p values (e.g., 20) are in line with G’s optimization of PSLR for practical purposes.

Utilizing the Fourier relationship between the autocorrelation function and the PSD, the vectorized form of the autocorrelation function can be expressed as

(9) $$r=A^{H}\left[\left(A\bar{s}\right)⊙{\left(A\bar{s}\right)}^{*}\right]$$

where the zero-padding version of s is as follows:

(10) $$\bar{s}={\left[s^{T}\;0_{1\times (M-1)}\right]}^{T}$$

has the same length of (2M−1) as the autocorrelation function. In addition, the functions of the (2M−1)×(2M−1) matrix A and $A^{H}$ are to realize the operation of the discrete Fourier transform (DFT) and the inverse discrete Fourier transform (IDFT), respectively. ⊙ is the Hadamard product.

Let

(11) $$J_{p}=\frac{{\left\|w_{\mathrm{SL}}⊙r\right\|}_{p}^{2}}{{\left\|w_{\mathrm{ML}}⊙r\right\|}_{p}^{2}}$$

(12) $$J_{1}=\lambda_{1}{\left({\left\|x+1\right\|}_{p_{2}}-\frac{3}{2}\right)}^{2}$$

(13) $$J_{2}=\lambda_{2}{\left({\left\|x-1\right\|}_{p_{2}}-\frac{3}{2}\right)}^{2}$$

Then, the discretized G cost function can be expressed as

(14) $$\begin{array}{cc} J= & J_{p}+J_{1}+J_{2}\\ = & \frac{{\left\|w_{\mathrm{SL}}⊙r\right\|}_{p}^{2}}{{\left\|w_{\mathrm{ML}}⊙r\right\|}_{p}^{2}}+\lambda_{1}{\left({\left\|x+1\right\|}_{p_{2}}-\frac{3}{2}\right)}^{2}+\lambda_{2}{\left({\left\|x-1\right\|}_{p_{2}}-\frac{3}{2}\right)}^{2} \end{array}$$

where r is an autocorrelation vector of length $\left(2M-1\right)$, while $w_{\mathrm{SL}}$ and $w_{\mathrm{ML}}$ are vectors of length $\left(2M-1\right)$ composed of zeros and ones used to select the mainlobe rigions and sidelobe rigions, respectively. The mainlobe range of $w_{\mathrm{ML}}$ is ones, and the sidelobe range of $w_{\mathrm{SL}}$ is ones. For the initial waveform of an LFM, the mainlobe region consists of (2*K* − 1) points at the center of the autocorrelation function. For the initial waveform of an NLFM, the mainlobe region needs to be analyzed specifically based on the autocorrelation function of the initial waveform. ${\left\|•\right\|}_{p}$ refers to the discrete p-norm, while ${\left\|•\right\|}_{p_{2}}$ refers to the discrete $p_{2}$–norm, where $p_{2}$ is a finite but relatively large value. $\lambda_{1}$ is the penalty coefficient of $J_{1}$, and $\lambda_{2}$ is the penalty coefficient of $J_{2}$. The penalty function $J_{1}$ ensures that the maximum value of the vector x is 0.5, while the penalty function $J_{2}$ guarantees that the minimum value of the vector x is −0.5.

For the discrete G cost function in (14), we now want to calculate its gradient relative to x, which is a vector of length N. The operator can be expressed as

(15) $$\nabla_{x}={\left[\frac{\partial }{\partial x_{1}}\;\frac{\partial }{\partial x_{2}}\;\ldots \;\frac{\partial }{\partial x_{N}}\right]}^{T}$$

When (15) is applied to $J_{p}$ in (11), the gradient of $J_{p}$ is [30]

(16) $$\nabla_{x}J_{p}=4J_{p}{\bar{B}}^{T}\times ℑ\left\{{\bar{s}}^{*}\right.⊙\left(A^{H}\left[A\left(\left[\frac{W_{\mathrm{SL}}}{W_{\mathrm{SL}}^{T}{\left|r\right|}^{p}}-\frac{W_{\mathrm{ML}}}{W_{\mathrm{ML}}^{T}{\left|r\right|}^{p}}\right]\right.\right.\right.\left.\left.\left.\left.⊙{\left|r\right|}^{\left(p-2\right)}⊙r\right)⊙\left(A\bar{s}\right)\right]\right)\right\}•B$$

where $\bar{B}$ in (16) can be written as

(17) $$\bar{B}={\left[B^{T}\;0_{N\times (M-1)}\right]}^{T}$$

In (16), $ℑ\left\{•\right\}$ is used to extract the imaginary part of the variable, while $\left|•\right|$ computes the modulus of each element in the vector variable. The gradient in (16) can be calculated using only FFT/IFFT and matrix/vector multiplication, allowing for the efficient and rapid computation of optimization.

When (15) is applied to $J_{1}$ in (12), the gradient of $J_{1}$ is

(18) $$\nabla_{x}J_{1}=2\lambda_{1}({\left\|x+1\right\|}_{p_{2}}-\frac{3}{2})•{\left|x+1\right|}^{\left(1-p_{2}\right)}⊙{\left|x+1\right|}^{\left(p_{2}-2\right)}⊙\left(x+1\right)$$

In the same way, the gradient expression for $J_{2}$ is

(19) $$\nabla_{x}J_{2}=2\lambda_{2}({\left\|x-1\right\|}_{p_{2}}-\frac{3}{2})•{\left|x-1\right|}^{\left(1-p_{2}\right)}⊙{\left|x-1\right|}^{\left(p_{2}-2\right)}⊙\left(x-1\right)$$

Then, the gradient of the cost function of G is

(20) $$\nabla_{x}J=\nabla_{x}J_{p}+\nabla_{x}J_{1}+\nabla_{x}J_{2}$$

The optimization method of gradient descent involves iteratively updating the vector in the direction of the gradient descent, thereby reducing the objective function. The algorithm steps for the HFPFM-coded waveform based on the G gradient descent optimization method are presented in Algorithm 1. Here, N=L(BT) represents the number of frequency values encoded, while M=K(BT) denotes the number of points in the optimized waveform. The stopping criterion for gradient descent is either reaching the maximum number of iterations set or the 2-norm of the gradient of the cost function reaching the minimum gradient threshold, as shown in row 13 of Algorithm 1.
**Algorithm 1**. G gradient optimization of HFPFM waveform1:
Initialize: *M*, *BT*, *N*, *B*, *T*, *L*, *β*, *μ*, *ρ*_*up*_, *ρ*_*down*_, *c*, *p*, *λ*_1_, *λ*_2_
2:Repeat3:      $\mathrm{Evaluate}:\;J\left(x_{i-1}\right)$$\mathrm{and}\;\nabla_{x}\;J\left(x_{i-1}\right)$ via (11) and (17) 4:    
$q_{i}={-\nabla }_{x}\;J\left(x_{i-1}\right)$
$+{\beta q}_{i-1}$
5:      
$\mathrm{If}\;(\nabla_{x}J\left(x_{i-1}\right){)}^{T}q_{i}\geq 0$
6:            
$q_{i}=-\nabla_{x}\;J\left(x_{i-1}\right)$
7:      End (If)8:      While
$\;J\left(x_{i-1}+{\mu q}_{i}\right)>J\left(x_{i-1}\right)+c\mu (\nabla_{x}\;J\left(x_{i-1}\right){)}^{T}\;q_{i}$
9:            
$\mu =\rho_{down}\mu$
10:      End (While)11:      
$x_{i}=x_{i-1}+{\mu q}_{i}\;,\;\mu =\rho_{up}\mu$
12:     
 i=i+1
13:$\mathrm{Until}\;i\;=I\;or\;\left\|\nabla_{x}J\left(x_{i}\right)\right\|<g_{min}$

## 3. Accomplishment of Optimization Model

In this section, we optimized the waveform using the model introduced in Section 2, exploring the impact of different values of p on the optimization results and evaluating the optimized waveforms. Firstly, we present the optimization results for the LFM waveform used as the initial waveform. Secondly, we demonstrate the optimization results for the NLFM waveform as the initial waveform. Finally, we obtained a waveform with extremely low sidelobes.

### 3.1. The Impact of the Value of p on the Results

LFM waveforms are the most common frequency modulation waveforms. Here, we set M to 2048, L to 2, β to 0.95, initial step size μ to $1\times 10^{-4}$, step size increase factor $\rho_{up}$ to 1.01, step size reduction factor $\rho_{down}$ to 0.9, sufficient reduction parameter to c to make sure the objective function is reduced enough, to $1\;\times \;10^{-2}$, and minimum gradient threshold to $g_{\min}=1\times 10^{-6}$, and the maximum number of iterations was set to $1\times 10^{6}$. In order to achieve precise control over the spectrum range, the values of penalty coefficients $\lambda_{1}$ and $\lambda_{2}$ are also continuously changing. x is a vector of length *N*, representing the range of frequency points. When the LFM waveform is used as the initial waveform, x consists of *N* uniformly sampled points from −0.5 to 0.5.

Appendix A lists the optimized values of PSLR and ISLR, obtained using LFM waveforms as initial waveforms, for bandwidth-time products BT of 64, 128, 256, and 512. For each value of BT, the minimum ISLR value and the minimum PSLR value are highlighted in bold.

We can observe some trends in Appendix A. Firstly, the gradient descent method can effectively optimize LFM waveforms. Secondly, we can observe that as the p value increases, the ISLR values tend to increase, while the PSLR values tend to decrease (although the ISLR and PSLR values are not strictly monotonically decreasing or increasing, which may be a result of the limited precision effects in gradient computations). The minimum value of ISLR for the four scenarios occurs when p is 2, while the minimum value of PSLR occurs when p is 18 or 20. Therefore, we can conclude that smaller p values tend to provide better ISLR results, while larger p values tend to provide better PSLR results. Based on the observed trends, we can choose the value of p depending on whether the goal of optimization is more inclined toward PSLR or ISLR.

**Figure 2:**
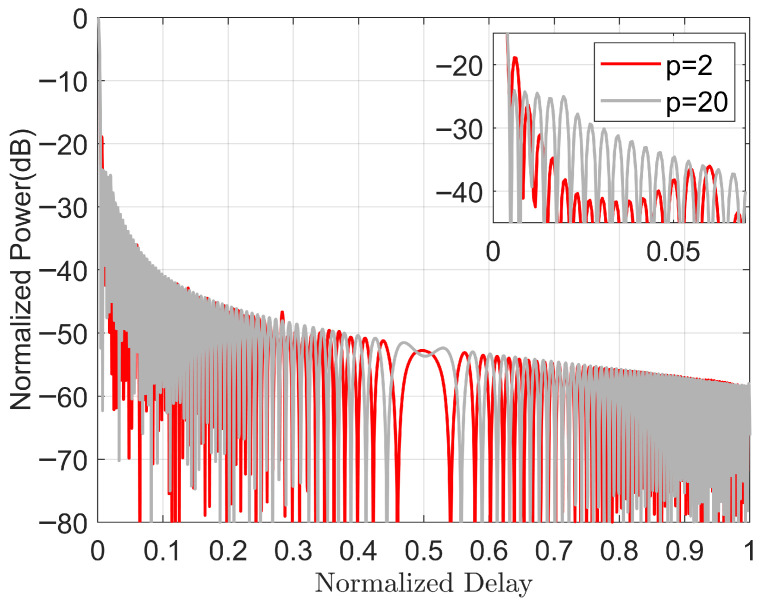
Autocorrelations of optimized HFPFM waveforms using LFM initialization with BT=256.

Having explored the large-scale trends, we now discuss some specific values to further explore properties such as autocorrelation function. We focus on the case of p = 2 ($\lambda_{1}$ = $\lambda_{2}$ = 1.13) and p = 20 ($\lambda_{1}$ = $\lambda_{2}$ = 0.3) at BT=256, as shown in Figure 2.

Figure 2 depicts the one-sided autocorrelation function in both cases. From this, we can see that there is a higher sidelobe peak at p = 2 (about 0.006 and 0.28 at the normalization delay). Overall, p = 2 has a lower sidelobe response (especially since it has a much lower sidelobe response at 0.01 to 0.05), which favors a lower ISLR value. Furthermore, from Figure 2, we can also see that the waveform is flatter with a lower PSLR (larger p-value) than when it has a lower ISLR.

### 3.2. Waveforms Performance Assessment

#### 3.2.1. Optimized Waveform Initialized with an LFM Waveform

**Figure 3:**
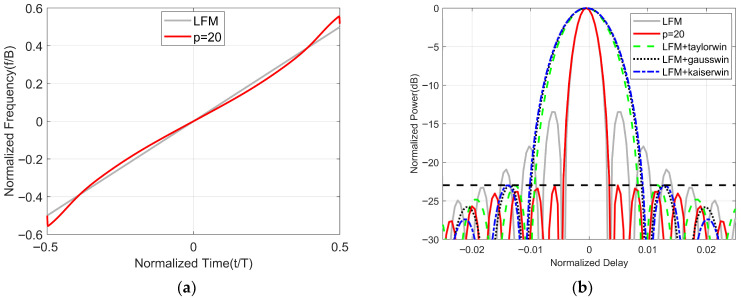
Optimized waveform initialized by LFM waveform. (**a**) Time–frequency structure. (**b**) Comparison of autocorrelation performance of different waveforms.

Here, we set B=300 MHz and T=20 μs. The normalized time-frequency structure of the optimized waveform (p=20) initialized with the LFM waveform is shown in Figure 3a. From this time–frequency structure, we can observe that the trend of the time–frequency curve of the optimized waveform is generally similar to that of the LFM waveform, except for some slight distortion at the beginning and end. The minimum value of the frequency approaches −B/2 and the maximum value approaches B/2, which is attributed to the constraints of the penalty function in Equation (14). More details of the mainlobe of the optimized waveform (p=20) initialized with the LFM waveform are shown in Figure 3b. As shown in Figure 3b, the optimized waveform has a PSLR of −22.9 dB, which is an improvement of 9 dB over the initial LFM waveform. Compared to the LFM waveform, the 3 dB mainlobe width of the optimized waveform has not been broadened, which is due to the constraints of $w_{\mathrm{SL}}$ and $w_{\mathrm{ML}}$ in Equation (14). That is to say, there is no loss of resolution. In Figure 3b, it can be observed that when the LFM is combined with the Taylor window, Gaussian window, or Kaiser window to achieve the same PSLR (indicated in Figure 3b by a black dashed line), the broadening of the mainlobe is larger than that of the optimized waveform.

In summary, we can conclude that the optimized waveform can reduce the level of sidelobes under the same mainlobe and achieve a smaller mainlobe under equivalent sidelobe levels. In other words, the optimized waveform possesses the excellent characteristics of high resolution and low sidelobes.

**Figure 4:**
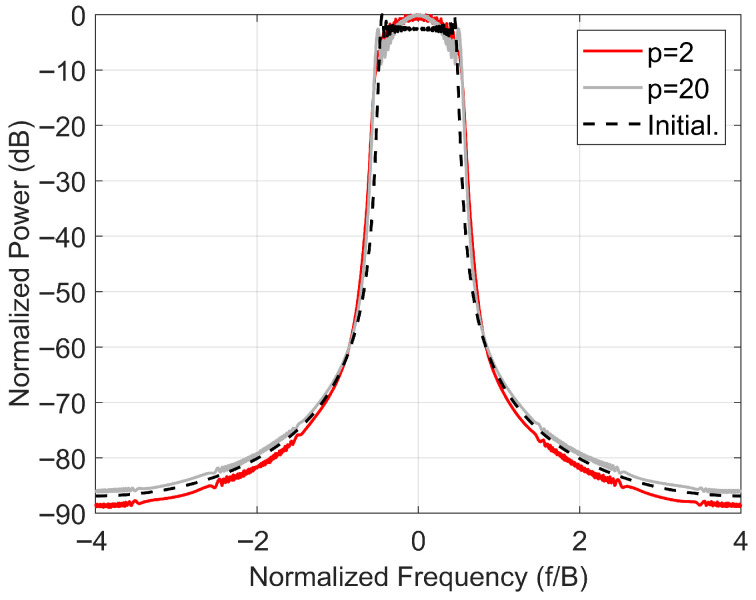
Power spectra of optimized HFPFM-coded waveforms for *p* = 2 and 20.

In addition, Figure 4 shows the power spectrum of the optimized waveform when the LFM waveform is used as the initial waveform. We can observe that the spectrum of the optimized waveform has not been broadened, which is also an advantage of HFPFM encoded waveforms: they possess precise spectrum control capabilities.

#### 3.2.2. Optimized Waveform Initialized with NLFM Waveform

**Figure 5:**
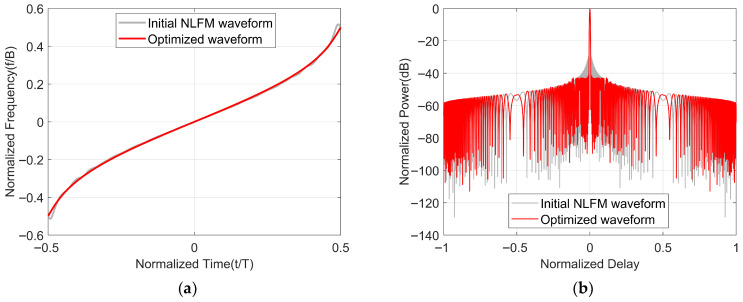
Optimized waveform initialized by NLFM waveform. (**a**) Time–frequency structure. (**b**) Comparison of autocorrelation performance of waveforms before and after optimization.

Next, we will optimize the NLFM waveform based on POSP as the initialized waveform, with the selected parameters of Taylor windows being $\bar{n}=4$, sll=−30, BT=1000, B=100 MHz, $\lambda_{1}$ = $\lambda_{2}$ = $5\times 10^{-3}$. When the NLFM waveform is used as the initial waveform, x consists of *N* non-uniformly sampled points from −0.5 to 0.5, as shown in Figure 5a. The instantaneous frequency function and autocorrelation of the waveform before and after optimization are shown in Figure 5. From Figure 5a, we can observe that the waveforms before and after optimization exhibit similar time–frequency relationships, with the optimized waveform displaying slight jitter at the beginning and end positions. From Figure 5b, it can be observed that the optimized waveform significantly reduces the value of PSLR.

**Figure 6:**
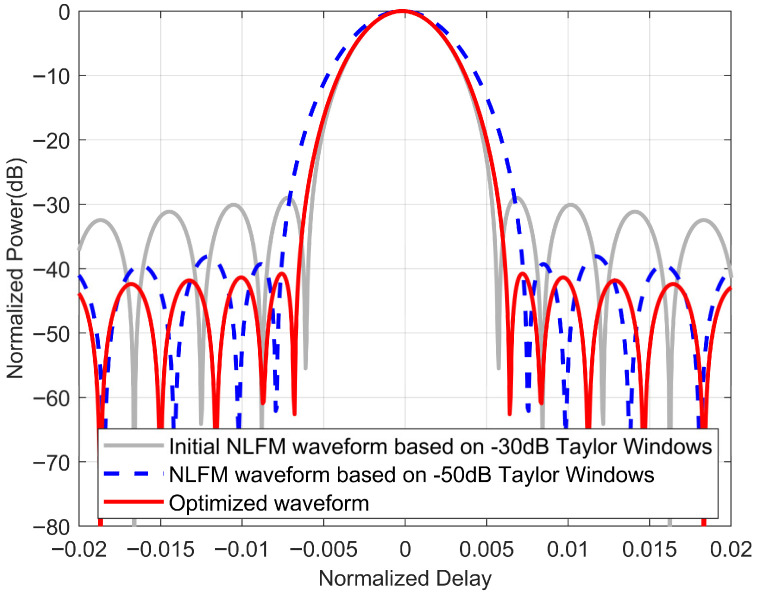
A close-up of the autocorrelation function of the NLFM waveform before and after optimization.

Furthermore, Figure 6 presents a close-up of the autocorrelation of the waveforms before and after optimization, which allows us to observe more intuitively: on one hand, the optimized waveform maintains the same 3 dB mainlobe width as the initial waveform while reducing the level of the sidelobes; on the other hand, at the same sidelobe level, the optimized waveform has a smaller mainlobe width compared to the NLFM waveform based on POSP.

The performance comparison of different waveforms is shown in Table 1, in which the impulse response width (IRW) refers to the 3 dB mainlobe width of the impulse response. When the LFM waveform is used as the initial waveform, firstly, compared to the LFM waveform, the optimized waveform can reduce the PSLR value by over 9 dB without widening the mainlobe width, which is superior to those presented in [28,31]. Secondly, compared to the LFM waveform with the Taylor window, Gaussian window, and Kaiser window, the optimized waveform can achieve a smaller mainlobe at the same sidelobe level, which is consistent with Figure 3b.

When the NLFM waveform is used as the initial waveform, it is observed that, firstly, the optimized waveform can reduce the PSLR value by more than 11 dB compared to the initial waveform. Secondly, compared to the NLFM waveforms generated by the Taylor window-based POSP method, the optimized waveform achieves a reduction in the 3 dB mainlobe width from 1.3125 to 1.1250, resulting in an equivalent improvement of nearly 15% in resolution without increasing the PSLR value. Furthermore, the performance of the optimized waveform surpasses that of the waveforms presented in [28,32].

In conclusion, the optimized waveform exhibits superior performance with a smaller mainlobe and lower sidelobes, indicating that the optimized waveform possesses the excellent characteristics of high resolution and low sidelobes.

**Figure 7:**
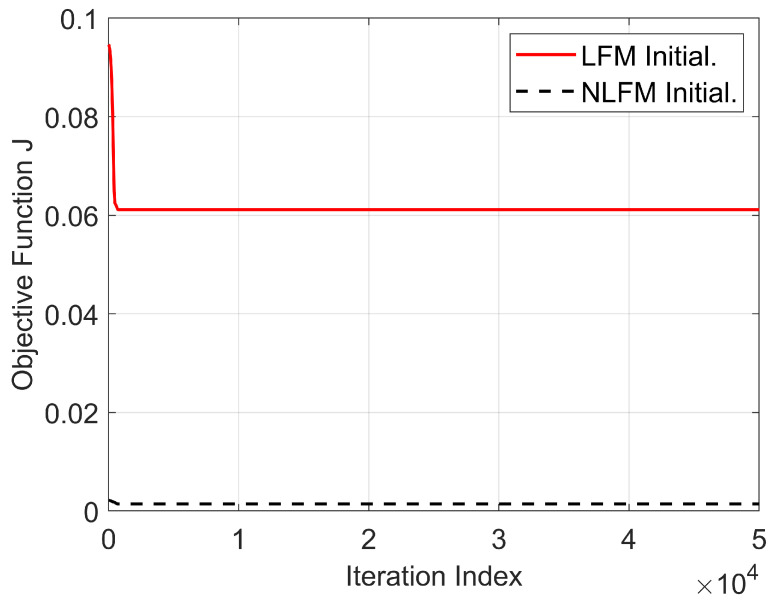
Convergence of the objective function *J* with the number of iterations.

Figure 7 shows the curve of the objective function J versus the number of iterations when LFM and NLFM waveforms are used as the initial signals. As can be seen from Figure 7, as the number of iterations increases, the objective functions J of both waveforms first decrease and then stabilize, reaching a convergent state. Both waveforms reach convergence at a relatively small number of iterations, which also demonstrates the efficiency of the algorithm proposed in this paper.

#### 3.2.3. System Test

**Figure 8:**
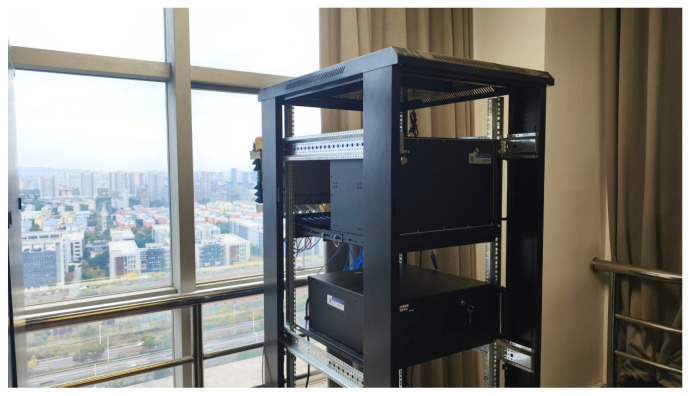
Photo of the equipment used for waveform measurement.

**Figure 9:**
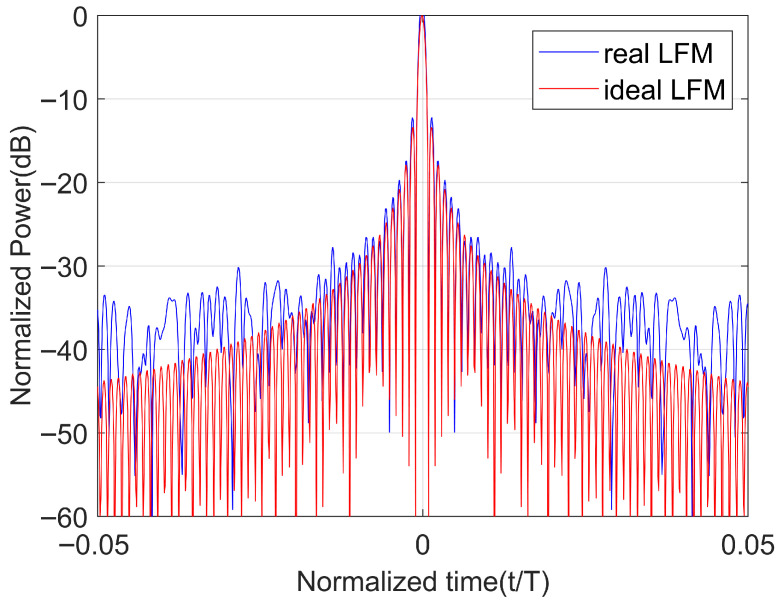
Comparison of the autocorrelation of ideal LFM waveform and real LFM waveform.

**Figure 10:**
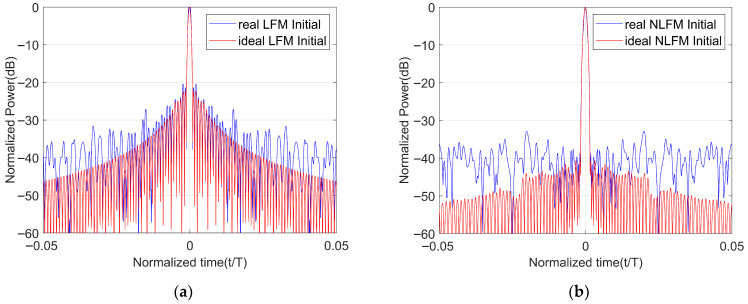
Comparison chart of the autocorrelation between the ideal optimized waveform and the real optimized waveform. (**a**) Comparison of the autocorrelation of ideal LFM optimized waveform and real LFM optimized waveform. (**b**) Comparison of the autocorrelation of the ideal NLFM optimized waveform and the real NLFM optimized waveform.

In order to reflect the performance of the transmitted waveform in an actual system, we conducted an experiment. We transmitted an LFM waveform, a signal optimized using the LFM waveform as the initial waveform, and a signal optimized using the NLFM waveform as the initial waveform. The tested equipment is shown in Figure 8. Figure 9 shows a comparison of the autocorrelation of the LFM waveform in the actual system and the ideal LFM waveform. Figure 10a shows a comparison of the autocorrelation of the LFM-optimized waveform in the actual system and the ideal LFM-optimized waveform. Figure 10b shows a comparison of the autocorrelation of the NLFM-optimized waveform in the actual system and the ideal NLFM-optimized waveform.

As can be seen from Figure 9 and Figure 10, the PSLR of the three waveforms in the actual system increased by 1.2103 dB, 1.8485 dB, and 10.0311 dB, respectively. So, the waveform we designed has a similar sensitivity to the actual system to that of the LFM waveform.

#### 3.2.4. Doppler Tolerance Assessment

**Figure 11:**
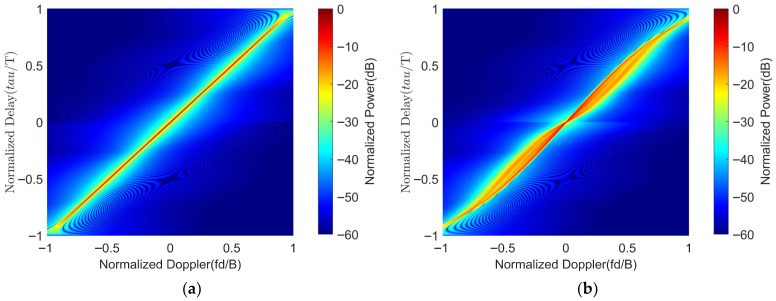
The ambiguity function of the LFM waveform and the optimized waveform. (**a**) The ambiguity function of the LFM waveform. (**b**) The ambiguity function of the optimized signal with the LFM waveform as the initial waveform.

The ambiguity function of the LFM waveform and the ambiguity function of the optimized signal with the LFM waveform as the initial waveform are shown in Figure 11.

**Figure 12:**
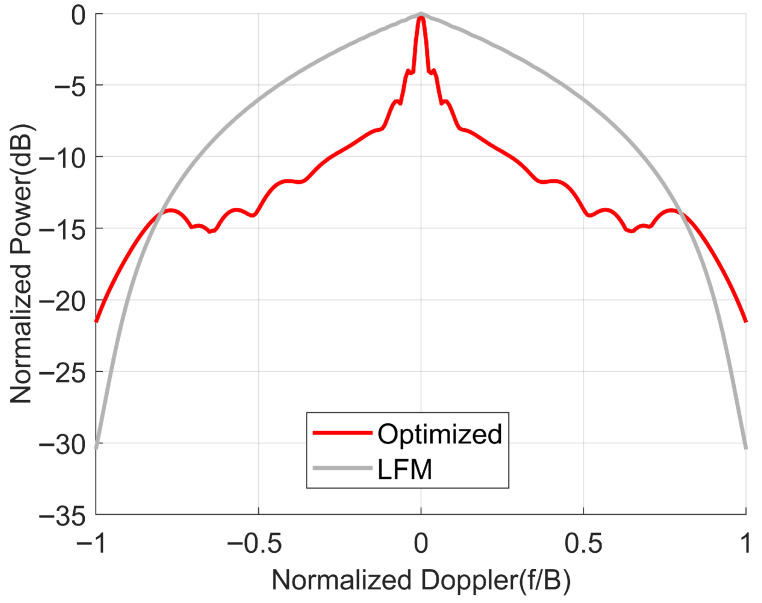
Ambiguity function peak value as a function of normalized Doppler.

We can further examine the level of Doppler tolerance by plotting the delay-Doppler ridge. Figure 12 shows the peak value of the Ambiguity function as a function of the normalized Doppler.

From Figure 12, we can see that due to the reduction in the sidelobes after optimization, the Doppler tolerance of the optimized waveform is worse compared to the LFM waveform. At this point, the 3 dB Doppler tolerance of the LFM waveform is 0.5819B (B is the bandwidth), which is 176.7 MHz. The 3 dB Doppler tolerance of the optimized waveform is 0.0396B, which is 11.88 MHz.

Take airborne SAR as an example. Suppose the aircraft’s flight speed is 120 m/s, the oblique angle is 45 degrees, and the carrier frequency is 10 GHz. At this point, the Doppler shift is about 5657 Hz, which is much smaller than the Doppler tolerance of the waveform we optimized. Therefore, although the optimized waveform has worse Doppler tolerance compared to the LFM waveform, this issue can be ignored for SAR.

For spaceborne SAR, because the satellite moves at a high speed, the Doppler shift can be quite significant. However, since the orbit of a spaceborne SAR is precisely known, its Doppler shift can be compensated for. Therefore, the impact of the Doppler shift can be considered negligible for spaceborne SAR.

#### 3.2.5. Ultra-Low Sidelobe Waveform Design

In order to design waveforms with extremely low sidelobes, we utilize the NLFM waveform generated by the POSP method based on the Gaussian window (α=4) as the initial waveform. The parameters of the optimization process are shown in Table 2.

**Figure 13:**
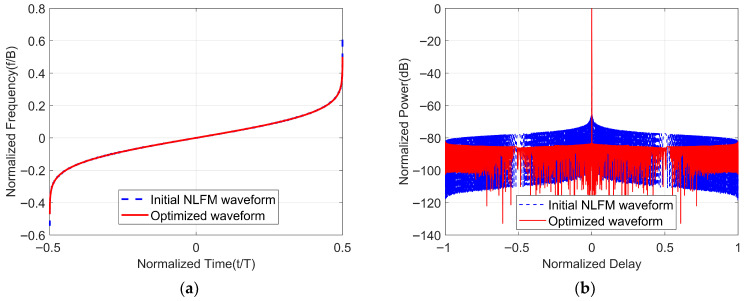
Ultra-low sidelobe optimized waveform initialized by NLFM waveform. (**a**) Time–frequency structure. (**b**) Comparison of the autocorrelation performance of waveforms before and after optimization.

**Figure 14:**
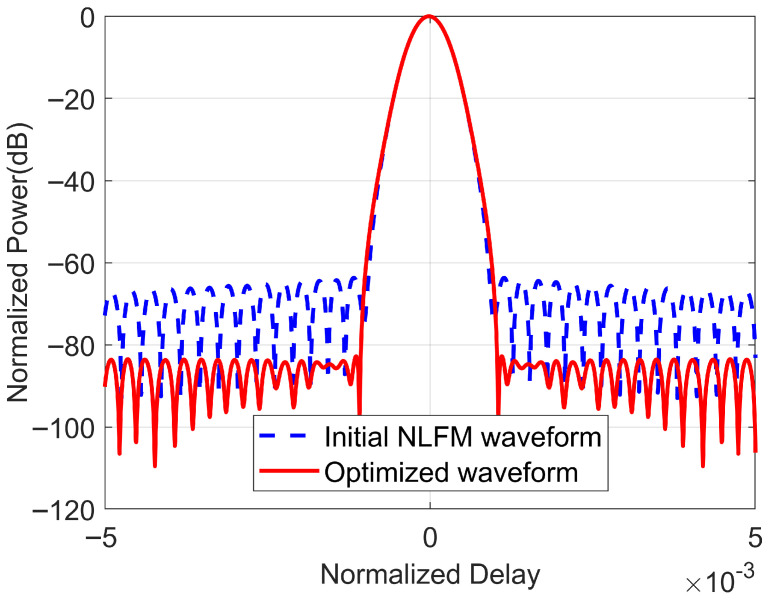
A close-up of the autocorrelation function of the waveform with extremely low sidelobes before and after optimization.

Figure 13 illustrates the time–frequency relationship and autocorrelation function of the waveform before and after optimization. Figure 14 presents the autocorrelation details (close-up) of the waveform before and after optimization. From Figure 14, it can be observed that the optimized waveform’s PSLR value decreases from −63.6 dB to −82.8 dB, which represents a reduction of over 19 dB. In this way, we achieved waveforms with extremely low sidelobes through optimization.

From Figure 14, we can see that the optimized waveform has the same 3-dB mainlobe width as the initial waveform and a lower PSLR value. Therefore, the waveform optimized through our proposed method possesses excellent characteristics of high resolution and low sidelobes.

## 4. SAR Point Target Imaging Simulation

In order to demonstrate the practical value of the high-resolution low-sidelobe waveform optimization method we proposed in actual SAR systems, we conducted point target simulation experiments. In this section, we will compare the waveform imaging results before and after optimization from Section 3.2.2 to verify that the optimized waveform has a stronger detection ability for weak targets, whereas the pre-optimization waveform may result in missed detections.

The point target imaging simulation utilizes a scenario with two targets, where the strong target is placed at the center of the scene and the weak target is positioned on one side at the same azimuth as the strong target but at a different range. The Radar Cross-Section (RCS) difference between the two point targets is 27 dB. The range-Doppler algorithm introduced in [1] was used for SAR point target imaging. We conducted point target imaging simulations using two different sets of waveforms: the first set employed NLFM waveforms based on the Taylor window ($\bar{n}=4$, sll=−30) for the range signal, and LFM waveforms with a Taylor window ($\bar{n}=4$, sll=−30) for the azimuth signal. The second set used optimized waveforms for the range signal, with the same LFM waveforms and Taylor window for the azimuth signal. Notably, the range signal in the second set is an optimized version of the range signal in the first set, while the azimuth signals for both sets are identical. The remaining parameter settings for the two-point target imaging simulations are identical. To make the point target simulation experiment more realistic, we added 30 dB of white noise to the distance signals in both experimental groups.

**Figure 15:**
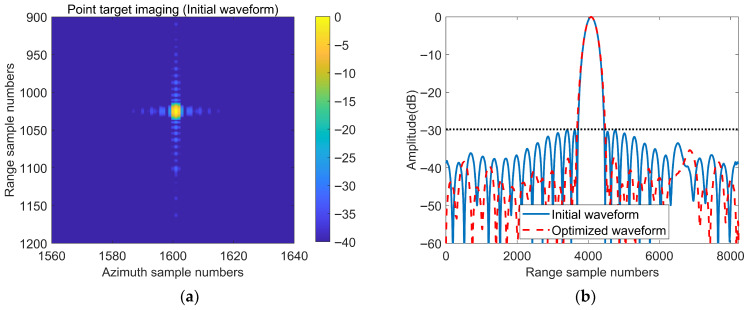
Point target imaging results of the initial waveform and the optimized waveform. (**a**) Point target imaging result of the initial waveform. (**b**) Point target imaging result of the optimized waveform.

**Figure 16:**
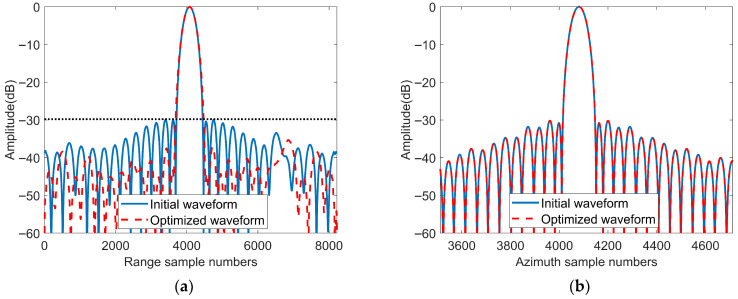
Range and azimuth profiles of the strong target shown in Figure 15. (**a**) Range profile of the strong target. (**b**) Azimuth profile of the strong target.

The imaging results are shown in Figure 15 and Figure 16. Figure 15 presents the imaging results after interpolation of the point targets. From Figure 15b, we can observe the imaging results of the weak targets (circled in red), whereas in Figure 15a, the weak targets are submerged in the sidelobes of the strong targets, resulting in missed detections. Figure 16 shows the range and azimuth profiles of the strong target. From the range profile shown in Figure 16a, we can observe that the optimized waveform clearly forms an image of the weak target to the right of the strong target, whereas the PSLR of the initial waveform is stronger than that of the weak target, making it unable to form an image of the weak target. The azimuth profiles of the initial waveform and the optimized waveform are the same. The simulation results of these point targets verify the effectiveness of the optimization method proposed in this article for weak target detection in practical SAR imaging.

## 5. Conclusions

In this paper, we propose a gradient descent optimization method for SAR systems based on an HFPFM coding model that generates waveform with lower sidelobes and a smaller mainlobe. The proposed optimization approach is based on the HFPFM coding model, which allows for precise control over the spectral range. Utilizing gradient descent, the method can simultaneously optimize all parameters and is efficiently implemented using only matrix multiplication and FFT/IFFT operations. We constructed an optimization objective function where different values of p make the optimization more inclined towards ISLR or PSLR. Through detailed experiments, we found that the optimized waveform, compared to LFM, can reduce PSLR by more than 9 dB without widening the mainlobe; compared to NLFM waveforms with the same PSLR, it has a smaller mainlobe width, resulting in an equivalent improvement of nearly 15% in resolution without increasing the PSLR value. Therefore, this method can produce high-resolution and low-sidelobe waveforms without compromising SNR. We also demonstrate the generation of a waveform with extremely low PSLR (−82.8 dB) using this method. Finally, through SAR point target imaging simulations, we validate the effectiveness of the optimized high-resolution low-sidelobe waveforms in weak target detection.

## Figures and Tables

**Table 1:** The performance comparison of different waveforms.

Waveform	IRW (Sample)	PSLR (dB)
LFM waveform	0.89	−13.3101
NLFM waveform based on POSP		
—Taylor window $(\bar{n}=4$, sll=−30)	1.0938	−28.9775
—Taylor window $(\bar{n}=4$, sll=−50)	1.3125	−38.0903
Proposed optimized waveform		
—Initialized by LFM waveform	0.89	−22.9607
—Initialized by NLFM waveform	1.1250	−40.7706
Optimized waveform in [28]		
—Initialized by LFM waveform	0.89	−18.51
—Initialized by NLFM waveform	1.17	−40.2
Waveform of LFM with Taylor window	1	−22.9336
Waveform of LFM with Gaussian window	1.0625	−22.9737
Waveform of LFM with Kaiser window	1.0859	−22.9332
Optimized NLFM waveform in [31], v = 0.9	0.9	−16.96
Iterative method in [32]	1.27	−40

**Table 2:** The parameters of the optimization process.

Parameter	Value	Parameter	Value
M	20,000	BT	5000
B	500 MHz	T	10 μs
L	2	K	4
*N*	10,000	α	4
$\lambda_{1}$	$1\times 10^{-7}$	$\lambda_{2}$	$1\times 10^{-7}$

## Data Availability

No new data were created or analyzed in this study. Data sharing is not applicable to this article.

## Abbreviations

The following abbreviations are used in this article:

SAR	Synthetic aperture radar
LFM	Linear frequency modulation
SNR	Signal-to-noise ratio
NLFM	Nonlinear frequency modulation
HFPFM	High Freedom Parameterized Frequency Modulation
FFT	Fast Fourier transform
IFFT	Inverse fast Fourier transform
PSLR	Peak sidelobe ratio
SVA	Spatially variant apodization
SOCP	Second-order cone programming
ROI	Regions of interest
CAN	Cyclic algorithm new
ISL	Integrated sidelobe level
CD	Coordinate descent
ADMM	Alternating direction method of multipliers
AF	Ambiguity function
MM	Majorization minimization
PSL	Peak sidelobe level
PSD	Power spectral density
POSP	Principle of stationary phase
PCFM	Polyphase-coded frequency modulation
FTE	Frequency template errors
ISLR	Integrated sidelobe level ratio
DFT	Discrete Fourier transform
IDFT	Inverse discrete Fourier transform
IRW	Impulse response width
RCS	Radar Cross-Section

## Appendix A

**Table A1:** ISLR for M=2048 and *L* = 2.

		N=L×BT			
		**128**	**256**	**512**	**1024**
Optimized G p-Norm	2	**−16.0294**	**−19.9178**	**−15.4038**	**−19.5948**
	3	−15.3018	−17.6281	−15.4023	−19.5874
	4	−15.0192	−16.7355	−15.1930	−18.1953
	5	−14.6524	−16.6370	−15.3473	−17.6102
	6	−14.5686	−16.4206	−15.4018	−17.1737
	7	−14.4271	−16.2745	−15.0557	−16.9181
	8	−14.5646	−16.1936	−15.3766	−16.4501
	9	−14.4803	−16.0495	−15.2623	−16.7973
	10	−14.8968	−16.0487	−15.1239	−16.4683
	11	−14.8253	−15.9870	−15.0355	−16.2706
	12	−14.6831	−15.8844	−14.8510	−16.1442
	13	−14.6911	−16.0493	−14.8621	−15.9762
	14	−14.2801	−15.9836	−14.9011	−15.7657
	15	−14.2840	−15.9979	−14.7522	−16.4101
	16	−14.7491	−15.9765	−14.8405	−16.2954
	17	−14.9782	−15.9599	−14.7995	−16.3314
	18	−14.9344	−15.9948	−14.2310	−16.2063
	19	−14.9358	−15.9125	−14.9075	−16.1102
	20	−14.9605	−15.9120	−14.9592	−16.7768

**Table A2:** PSLR for M=2048 and *L* = 2.

		N=L×BT			
		**128**	**256**	**512**	**1024**
Optimized G p-Norm	2	−19.3184	−22.9398	−17.2452	−21.3726
	3	−20.5021	−23.3675	−18.7390	−24.2558
	4	−21.3688	−23.5975	−19.9456	−24.0623
	5	−21.4145	−24.1332	−21.3076	−24.2986
	6	−21.7829	−24.3367	−22.1324	−24.5260
	7	−21.7729	−24.3448	−22.0493	−24.5008
	8	−22.0596	−24.4321	−22.7601	−24.1343
	9	−22.0208	−24.3288	−22.8403	−24.7533
	10	−22.9573	−24.4304	−22.7629	−24.3992
	11	−22.8565	−24.3873	−22.7313	−24.2288
	12	−22.5726	−24.2124	−22.5108	−24.1232
	13	−22.6759	−24.6112	−22.5337	−23.9172
	14	−22.0930	−24.6125	−22.6975	−23.6823
	15	−22.1262	−24.5854	−22.5018	−24.5190
	16	−22.8431	−24.5934	−22.6457	−24.4071
	17	−23.3960	−24.6129	−22.6170	−24.4765
	18	−23.2840	**−24.7336**	−21.8611	−24.3306
	19	−23.2965	−24.5900	−22.8458	−24.2161
	20	**−23.3463**	−24.6189	**−22.9607**	**−25.0383**
